# Systematic metabolic engineering for improvement of glycosylation efficiency in *Escherichia coli*

**DOI:** 10.1016/j.bbrc.2012.02.020

**Published:** 2012-03-16

**Authors:** Jagroop Pandhal, Pratik Desai, Caroline Walpole, Leyla Doroudi, Dmitry Malyshev, Phillip C. Wright

**Affiliations:** ChELSI Institute, Biological and Environmental Systems Group, Department of Chemical and Biological Engineering, University of Sheffield, Sheffield, UK

**Keywords:** Glycosylation, *Escherichia coli*, Efficiency, Proteomics, Selective reaction monitoring

## Abstract

Recently, efforts to increase the toolkit which *Escherichia coli* cells possess for recombinant protein production in industrial applications, has led to steady progress towards making glycosylated therapeutic proteins. Although the desire to make therapeutically relevant complex proteins with elaborate human-type glycans is a major goal, the relatively poor efficiency of the N-glycosylation process of foreign proteins in *E. coli* remains a hindrance for industry take-up. In this study, a systematic approach was used to increase glycoprotein production titres of an exemplar protein, AcrA, and the resulting glycosylation efficiency was quantified using a combination of Western blots and pseudo Selective Reaction Monitoring (pSRM). Western blot and pSRM results demonstrate that codon optimising the oligosaccharyltransferase, PglB, for *E. coli* expression, increases efficiency by 77% and 101%, respectively. Furthermore, increasing expression of glycosyltransferase, WecA, in *E. coli* improves efficiency by 43% and 27%, respectively. However, increasing the amount of donor lipid used in the glycosylation process did not impact on the glycosylation efficiency in this system, with this specific protein.

## Highlights

► *Escherichia coli* cells require modifying to make ideal hosts for producing glycoproteins. ► Codon optimising *pglB* leads to increased glycosylation efficiency. ► Lipid linked precursors do not appear to be limiting for *E. coli* N-glycosylation. ► Increasing expression of WecA improves *E. coli* N-glycosylation efficiency.

## 1 Introduction

The ambition of using bacteria to make complex post-translationally modified human protein therapeutics remains a major challenge for the bioprocessing community. The motivation is to use production systems that are less costly than mammalian expression systems with a higher level of final product control, i.e. little or no heterogeneity, is leading to exciting progress. Although expression of large functional complex proteins in *Escherichia coli*, present problems of their own, steady progress has been made [1]. The production of smaller therapeutic fragment proteins, working alone or intended for therapeutic fusion proteins, such as antibody fragments, has been demonstrated as an attractive alternative [2–4]. Advantages of small therapeutic proteins include flexibility in structure which increases binding possibilities and enhanced penetration of tissues [2]. A significant advantage is the reduced costs of production in *E. coli*. The ability to perform bacterial N-glycosylation of target proteins in *E. coli* has been demonstrated using the well characterised N-glycosylation pathway from *Campylobacter jejuni* [5–7]. Although performed in an exemplar protein, AcrA, sourced from *C. jejuni*, the process has been shown to be functional although very inefficient [6,8,9]. In previous work, the glycosylation efficiency, i.e. the percentage of glycosylated target protein compared to total target protein, of AcrA was reported to be just 13% [8]. It is thought that the occupancy rate of a protein with a specific sugar structure could be dependent on factors such as the site of the glycosylation consensus sequence and export pathways, but there is not a general rule [10–12]. The Pgl pathway is not native to *E. coli* and therefore metabolic constraints may also play a significant role in glycosylation efficiency. For this reason, an iterative metabolic engineering strategy was developed to identify bottlenecks and highlight potential targets to improve glycosylation efficiency [8]. A discovery-driven proteomics workflow, using chemical tagging was employed with a mixture model on graphs (MMG) approach [13] to suggest pathways that could be altered. An increase in expression of isocitrate lyase in the glycoxylate shunt resulted in an increase in glycosylation efficiency of AcrA by almost 3-fold to 48% [8].

Recently, Schwarz et al. [9] glycosylated AcrA and two human antibody fragments F8 and CH2, using a system where the native enzyme undecaprenyl-phosphate alpha-*N*-acetylglucosaminyl 1-phosphate transferase (WecA) in *E. coli* was used to add the initial sugar *N*-acetylglucosamine (GlcNAc), the same initial sugar found in human *N*-glycoproteins. They reported glycosylation efficiencies of 40% and 5% for the antibody fragments, respectively. The overall titres of recombinant protein were very low (personal communication-Flavio Schwarz) and therefore improvements are required in overall recombinant protein production as well as glycosylation efficiency. The research presented here was carried out to address the glycosylation efficiency issue.

**Fig. 1:**
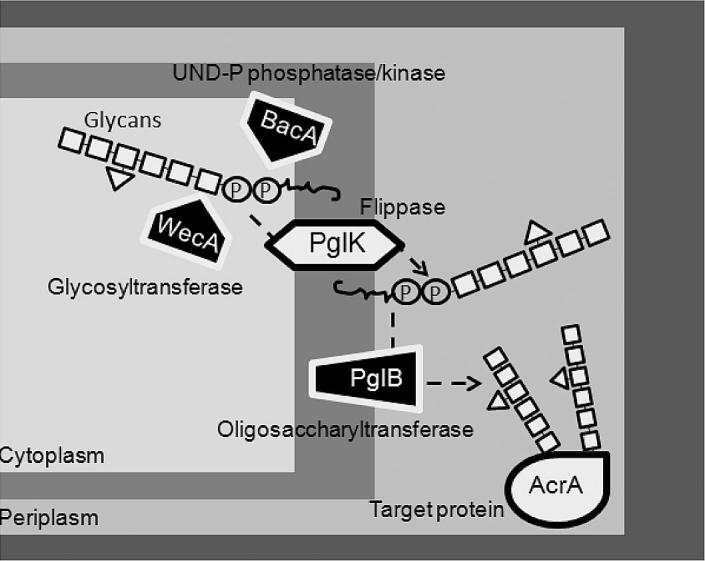
A schematic representation of an *E. coli* cell harbouring N-glycosylation capability. The systematic cellular engineering targets are shown as black boxes with white borders. The order of steps involved in the N-glycosylation can be followed using the black dashed arrows. Briefly, a lipid linked oligosaccharide is built in the cytoplasm using WecA to add the initial GlcNAc sugar to the phosphorylated lipid. This is transferred using PglK flippase to the periplasm, where the oligosaccharyltransferase, PglB, recognises the structure and transfers it onto the target protein (AcrA) on the appropriate consensus sequence. P = phosphate group.

In this work, the glycosylation efficiency of AcrA was tested using systematic genetic changes to the *E. coli* host cell system. A codon optimised oligosaccharyltransferase gene (*pglB*) was tested and quantified using Western blots and pseudo Selected Reaction Monitoring (pSRM) (see Pandhal et al. [8]). Western blots give an actual glycosylation efficiency percentage whereas pSRM confirms this value by providing fold difference changes in glycoprotein normalised by total protein. The effect of increasing WecA expression was also tested, as this glycosyltransferase recognises GlcNAc and attaches the sugar to asparagine residues [14]. Finally, the increase in expression of BacA was also tested. In this system, glycosylation involves the transfer of a heptasaccharide from an undecaprenyl-pyrophosphate donor to the asparagine side chain of proteins. BacA confers undecaprenyl pyrophosphate phosphatase activity [15], which potentially results in increased availability of undecaprenyl phosphate for glycoprotein synthesis. An increase in glycosylation efficiency from increased BacA presence would indicate this as a bottleneck in this system. The targets for engineering cells are shown in Fig. 1.

## 2 Materials and methods

All materials were purchased from Sigma–Aldrich (Dorset, U.K.) unless otherwise stated.

### 2.1 DNA cloning, PCR, mutagenesis and vectors

The chloramphenicol resistant vectors pACYC*pgl* [7] and pACYC*pgl2* [9] were implemented here to perform the N-glycosylation process. *PglB* is present on both vectors, but was removed in pACYC*pgl* to create pACYC*pgl* Δ*pglB*. Briefly, pACYC*pgl* was digested with BaeI (NEB, Herfordshire, UK) at 37 °C for 1 h. After confirmation by agarose gel electrophoresis, the linear DNA was digested with exonuclease BAL-31 (NEB) for 5, 10, 20 and 30 min at 30 °C. The reaction was stopped by addition of SureClean reagent (Bioline, London, UK). Overhangs were filled in using Phusion polymerase (NEB) following the manufacturer’s instructions. After further clean-up using SureClean reagent, vectors from all four time points were re-ligated using T4 DNA ligase (NEB) with manufacturer’s instructions for one hour at 37 °C. Plasmids were transformed into NEB5alpha competent cells (NEB) and a colony screen (forward primer GTGATAAAAATCCTATTCTC, reverse primer ACGCGATGCTTTGAAATATT) was performed. Briefly, sterile pipette tips were used to transfer colonies into 5 μL water for subsequent PCR and also to re-streak colonies on fresh LB agar (with antibiotics). The PCR program was as follows: 95 °C for 5 min, and 30 cycles of 95 °C for 30 s, annealing at 55 °C for 30 s and extension at 72 °C for times depending on the expected insert size. A final extension step of 72 °C for 10 min was included. Following agarose gel electrophoresis, PCR products that visually looked reduced in size were sent for sequencing at the Core Genome Facility (University of Sheffield). Plasmids with removed *pglB* were further tested phenotypically using Western blots to see if glycosylation was abolished (Western blot in Supplementary materials).

A codon optimised *pglB* gene was synthesised by DNA 2.0 (CA, USA) using the GeneDesigner software algorithm. The codon optimised sequence is given in the Supplementary materials, with screenshots of how the software is used. The gene was provided on vector pjexpress401 with kanamycin resistance and named pjexpress401*pglB*^∗^.

WecA was amplified from *E. coli* K12 DNA using the primer sequences presented in the Supplementary materials. PCR was performed as described previously [8], but with an annealing temperature of 57 °C. The amplified gene was purified using SureClean reagent and digested with restriction enzymes BamHI and XmaI for 1 h at 37 °C. The same digestion was performed on vector pjexpress401*pglB*^∗^. The vector backbone was purified using a gel extraction kit (Zymoresearch, Cambridge) and ligated to *wecA* using T4 DNA ligase. The vector was named pjexpress401*wecA.* This procedure was repeated using *bacA* amplified from *E. coli* K12 DNA using primers given in the Supplementary materials. However, BamHI and XbaI digestion was used prior to ligation. This vector was named pjexpress401*bacA.* The native (non-codon optimised) *pglB* gene was also amplified from the original pACYC*pgl* vector and inserted into pjexpress401 to make pjexpress401*pglB* using a similar procedure described above. This vector was used to re-introduce pglB activity to cells to test pACYC*pgl*Δ*pglB* mutation and serve as a control of pjexpress401*pglB*^∗^.

### 2.2 Cell growth, induction and harvesting

*E. coli* CLM24 cells [5] were used as host cells for this study. Ampicilin, kanamycin and chloramphenicol concentrations of 50 μg/ml were used where appropriate. Overnight cultures were used to seed 500 ml of LB-broth and grown at 37 °C with shaking at 180 rpm. When the optical density (O.D.) at 600 nm reached 0.5, cells were induced with 0.2% l-arabinose and 20 μM IPTG. 20 O.D. units worth of cells were harvested 3 h post induction via centrifugation at 10,000*g* for 15 min at 4 °C.

### 2.3 Protein purification

Proteins from the periplasm were prepped and AcrA purified using his-tag purification as described previously [8]. The resulting his-tag purification was prepared for quantitation using pSRM and Western blotting [8].

### 2.4 Western blots

Western blots were performed as described previously [8], except for alterations described briefly below. His-tag purified protein samples were quantified using RC/DC assay® (BioRad, UK) and 5 μg of total protein was loaded in each well (in 15 μL) combined with 4× loading buffer (1 M Tris–HCl(pH 6.8), 20% glycerol, 4% SDS (w/v), 0.1% (w/v) bromophenol blue (w/v), 10% beta-mercaptoethanol) and loaded onto SDS–PAGE gels. After transfer to nitrocellulose membrane using an iBlot® Dry Blotting System (Life Technologies, CA, USA), membranes were probed using 0.5 μL anti-C-terminal his-tag antibody (Sigma see manufacturer’s instructions). Detection was performed using chemiluminescence with Immobilin® horseradish peroxidase (HRP) substrate (Millipore, Watford, UK). Western blots were visualised and quantified using VisionWorks® LS (Cambridge, UK). Glycosylation efficiency was calculated as a percentage using intensity of glycoprotein over intensity of total (glycosylated and aglycosylated) protein.

### 2.5 pseudo Selective Reaction Monitoring (pSRM)

Samples were prepared for MS analysis by in-gel trypsin digestion as described previously [8]. After peptide extraction, the samples were dried and resuspended in buffer I (3% acetonitrile, 0.1% formic acid) ready for targeted MS quantitation. Briefly, an HCT Ultra PTM discovery ESI-Ion Trap MS/MS (Bruker Daltonics, Coventry UK) was used to perform high selectivity pSRM measurements of target peptides. The MS was operated in Ultrascan mode with a 3 *m*/*z* window. Ion accumulation was set to 180,000 with accumulation maximum of 200 ms. The intact masses and fragmentation transitions were monitored with 3 microscan averages. Reverse phase separation of peptides was achieved online using a microflow Ultimate 3000 LC system (Dionex, Surrey, U.K.) with 5% buffer II (97% acetonitrile, 0.1% formic acids) for 10 min followed by an increment to 40% buffer II for 30 min. A 90% buffer B for 10 min was then followed by a final return to 5% buffer B for 10 min.

The pSRM scans were analysed using DataAnalysis v4.0 (BrukerDaltonics, Coventry UK). The mean peak area ratios of the three technical replicates runs for each sample was compared to the control for each phenotype and relative values were recorded. This was repeated for biological replicate samples. Changes in glycosylation efficiency were calculated by normalising glycopeptide relative changes to total protein changes (calculated from aglycosylated peptide intensities) and shown as a percentage.

## 3 Results and discussion

The growth rates of engineered *E. coli* CLM24 cells were not compromised significantly compared to the control cells (see Supplementary materials). The Western blot analysis had the advantage of calculating an actual glycosylation efficiency value by comparing glycosylated to aglycosylated band intensity. Although pSRM was limited to revealing a fold change in glycosylation efficiency only, variation across biological replicates was lower using pSRM compared to Western blot analysis (10% compared to 30% respectively). The average technical variation using pSRM was 19%. Table 1 shows the pSRM peptide targets and elution times. Example spectra are given in the Supplementary materials.

Codon optimised oligosaccharyltransferase PglB was compared against standard PglB using pjexpress401 vectors and pACYC*pgl*Δ*pglB*, where the native *pglB* gene had been deleted. Cells were induced to express both versions of oligosaccharyltransferase as well as the target glycoprotein AcrA. In order to confirm the deletion of the *pglB* gene from pACYC*pgl*, *E. coli* CLM24 cells were induced to produce AcrA with the vectors pEC*acrA* and pACYC*pgl*Δ*pglB*. Western blot analysis showed the presence of one band, and therefore indicated that only aglycosylated AcrA was present (see Supplementary materials). This confirmed the deletion, as PglB is not present to transfer the glycan onto the protein and hence no glycoprotein is detected. These cells were further transformed with either (i) vector pjexpress401*pglB* or (ii) vector pjexpress401*pglB*^∗^. The resulting Western blot analysis shows multiple bands indicative of modified AcrA protein (mono and di-glycosylated) (see Supplementary materials).

**Fig. 2:**
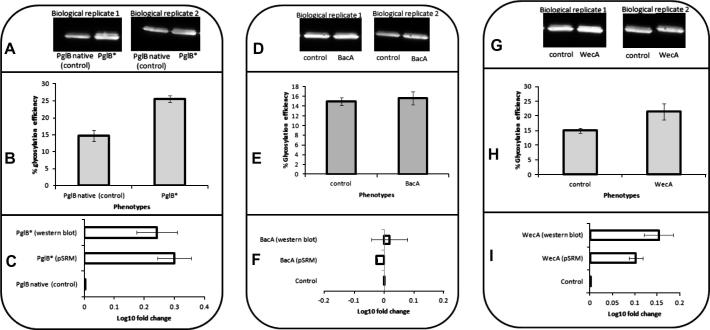
(A–C) A comparison of glycosylation efficiency in cells expressing native *pglB* from *C. jejuni* (control) and codon optimised PglB (PglB^∗^). (A) Western blot image. (B) Glycosylation efficiency calculated from Western blot images in part A. (C) Log 10 fold change in glycosylation efficiency calculated by Western blots in part A and pSRM. (D–F) A comparison of glycosylation efficiency in cells over expressing *bacA* to control cells. (D) Western blot image. (B) Glycosylation efficiency calculated from Western blot images in part D. (E) Log 10 fold change in glycosylation efficiency calculated by Western blots in part D and pSRM. (G–I) A comparison of glycosylation efficiency in cells over expressing *wecA* to control cells (G) Western blot image. (H) Glycosylation efficiency calculated from Western blot images in part G. (C) Log 10 fold change in glycosylation efficiency calculated by Western blots in part G and pSRM. The purified AcrA protein was probed with anti-C-terminal histidine antibody.

Fig. 2A–C shows Western blot and pSRM quantifications of AcrA from *E. coli* CLM24 cells containing pACYC*pgl*Δ*pglB* and supplemented with (i) pjexpress401*pglB* and (ii) pjexpress401*pglB*^∗^. Quantifications of the Western blot show an overall increase in glycosylation efficiency of 77 ± 27% in cells with codon optimised *pglB* compared to non-codon optimised *pglB*. pSRM results show a 101 ± 26% increase in efficiency when *pglB* is codon optimised. These results imply that improved expression levels of *pglB* in the glycosylation pathway could be a bottleneck in the production of glycosylated AcrA in *E. coli* cells and an increase in glycosylation efficiency is evident by both Western blot and pSRM.

The BacA enzyme, native to *E. coli*, harbours undecaprenyl pyrophosphate phosphatase activity through conversion of undecaprenyl pyrophosphate (UND-PP) to undecaprenyl phosphate (UND-P) [15]. This product is a key lipid intermediate involved in the synthesis of cell wall polymers such as peptidoglycan. The UND-P lipid is also required as a donor to add the attached heptasaccharide sugar (donated from sugar nucleotide donors) to the glycosylation consensus sequence on AcrA (D/E-Z-N-X-S/T, where X and Z can be any amino acid except for proline). Therefore, an increase in BacA would potentially increase the donor availability and increase glycosylation efficiency. Fig. 2D–F shows quantifications of the Western blot image and pSRM of *E. coli* CLM24 cells with pEC*acrA*, pACYC*pgl* and either a pjexpress401 control plasmid or pjexpress401*bacA*. Quantifications using both methods show no significant change in glycosylation efficiency. It is therefore presumed that undecaprenyl phosphate availability is not a glycosylation limiting component in the *E. coli* system.

WecA protein is native to *E. coli* and involved in the initiation of lipopolysaccharide synthesis [16]. This sugar transferase catalyses the transfer of GlcNAc-1-phosphate onto undecaprenyl phosphate (UND-P) to form UND-PP-GlcNAc. This protein can also transfer the GlcNAc onto the UND-P as the initial sugar of the *Campylobacter* heptasaccharide rather than the rare sugar bacillosamine. This new sugar structure is recognised by PglK and PglB in the glycosylation process [5,17]. Mass spectral evidence of the different glycan structure has been reported previously [7], although the abundance difference of the two different types has not. Subsequently, a pACYC*pgl2* vector has been created in which the pgl pathway was modified by deletion of *pglD*, *pglE*, *pglC*, *pglF* and *pglI* [9]. The new glycan is a hexasaccharide of GlcNAc (GalNAc)_6_ (glucose is also not added). In order to improve AcrA glycoprotein expression, WecA synthesis was induced using the pjexpress401*wecA* vector and the amount of glycoprotein with the new hexasaccharide structure was quantified and compared to control cells. The Western blot quantifications shown in Fig. 2G and H confirm a 43 ± 11% increase in glycosylation efficiency. pSRM calculations show a 27 ± 5% in glycosylation efficiency. This increase, although small, shows that WecA is a potential bottleneck for glycoprotein production in the *E. coli* system. Increasing its expression level further through IPTG induction, without comprising growth rate and AcrA production, could improve this further.

The aim of using *E. coli* cells to economically produce therapeutically relevant, glycosylated human protein therapeutics, requires generating a strain which harbours the highest efficiency of post-translationally modified target proteins. Metabolic engineering of cells to improve the amount of protein which carry the specific glycans is one approach, and progress has been made in this area by identifying targets using proteomics and metabolic network analysis. This study involved targeting specific metabolic enzymes and quantifying glycosylation efficiency using Western blots and pSRM.

The percentage of glycosylated recombinant AcrA protein, produced in *E. coli*, was increased by improving expression levels of oligosaccharyltransferase, *pglB*. The expression level was improved by codon optimisation and translated to a 77%and 101% increase in glycosylation efficiency (using Western blots and pSRM, respectively). The hypothesis that lipid linked precursor could also be a bottleneck was tested by increasing expression of *bacA*, which has undecaprenyl pyrophosphate phosphatase activity. However, Western blot analysis and pSRM quantification provided evidence that the hypothesis was unfounded with AcrA protein. The use of the *E. coli* native enzyme WecA to add the same glycan as present in human proteins, GlcNAc, was improved by increasing its expression. The modest increase in efficiency however (43% and 27%), implies that *E. coli* cells would have to be modified in tandem with other cellular engineering strategies in order to produce an ideal host for human therapeutic protein production.

## Figures and Tables

**Table 1:** pSRM targets. The following peptide *m*/*z* values were programmed into the HCT Ultra MRM software system.

Peptide	Modification⁎	Precursor mass	*m*/*z*	Transition ions	Retention time (min)
NGFKVPQIGVK	N/A	1185.7	396.2	416, 641	28.6
LYFIDSVIDANSGTVK	N/A	1742.0	871.5	791, 904	36.4
AVFDNN⁎NSTLLPGAFATITSEGFIQK	Bac(GalNAc)_5_Glc	4161.1	1041.4	1696, 1798	39.9
ATFENASKDFN⁎R	GlcNAc(GalNAc)_5_	2779.5	927.7	1188, 1289	23.6

⁎ Bac: bacillosamine, GalNAc: *N*-acetylgalactosamine, GlcNAc: *N*-acetylglucosamine, Glc: glucose.

## Appendix A Supplementary data

Supplementary data 1 The document containing supplementary material.
