# Supplementary material for: Systematic metabolic engineering for improvement of glycosylation efficiency in Escherichia coli
Source: Biochem Biophys Res Commun. 2012 Mar 16;419(3):40–3. doi: 10.1016/j.bbrc.2012.02.020 (PMC3401370; doi:10.1016/j.bbrc.2012.02.020)

Supplementary files


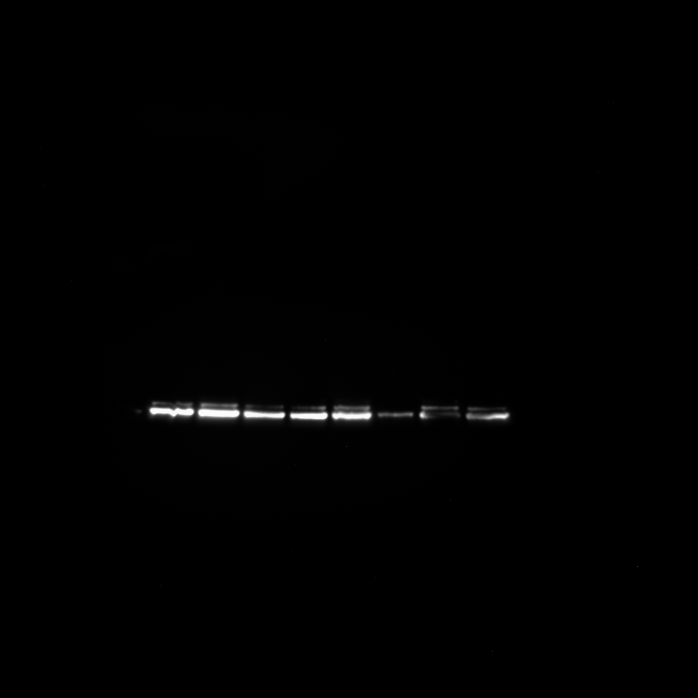
Western blot analysis

Lane 1 Lane 2 Lane 3

Di-glycosylated AcrA

Mono-glycosylated AcrA

Aglycosylated AcrA

Figure 1. A western blot of AcrA protein produced in *E. coli* CLM24 cells, purified by his-tag chromatography and blotted using a C-term anti-his antibody (Sigma Aldrich-Dorset, U.K.).Lane 1: *E. coli* CLM24 pEC*AcrA*; pACYC*pgl* Δ*pglB.* Lane 2: *E. coli* CLM24 pEC*AcrA*; pACYC*pgl* Δ*pglB*; pjexpress401*pglB*. Lane 3: *E. coli* CLM24 pEC*AcrA*; pACYC*pgl* (control).The absence of glycosylation is evident in lane 1 where only one band is visible (aglycosylated AcrA).

Codon optimised *pglB* using GeneDesigner software algorithm

ATGATTATCCTGGCCTACGTCTTTAGCGTTTTCTGTCGCTTTTACTGGGTTTGGTGGGCCTCT GAGTTCAACGAGTATTTCTTCAATAACCAGCTGATGATTATCAGCAATGATGGTTACGCATTCGCAGAAG GTGCGCGCGATATGATCGCAGGTTTCCATCAGCCGAATGATCTGTCCTACTATGGTTCCAGCCTGTCCGC

GTTGACGTACTGGCTGTACAAGATCACCCCGTTTAGCTTCGAATCCATTATCCTGTATATGAGCACCTTC

CTGAGCAGCTTGGTTGTTATTCCGACGATTCTGCTGGCTAATGAGTATAAGCGTCCGCTGATGGGTTTTG

TCGCAGCGCTGTTGGCGAGCATTGCAAACAGCTACTACAATCGTACTATGAGCGGTTACTATGACACCGA

TATGTTGGTTATTGTTCTGCCAATGTTTATCCTGTTCTTTATGGTGCGTATGATCCTGAAGAAAGATTTC

TTTTCTCTGATCGCGCTGCCGTTGTTCATCGGTATCTATCTGTGGTGGTATCCGTCTAGCTATACCCTGA

ACGTGGCCCTGATTGGTCTGTTTCTGATTTACACCCTGATCTTTCACCGTAAAGAAAAGATTTTCTATAT

CGCGGTGATCCTGAGCTCCCTGACGCTGTCCAACATTGCCTGGTTCTACCAGTCCGCGATTATCGTCATT

CTGTTTGCGCTGTTTGCTCTGGAGCAAAAACGCCTGAACTTTATGATCATTGGTATTCTGGGTAGCGCGA

CTCTGATCTTCCTGATTCTGAGCGGTGGCGTTGATCCAATCCTGTACCAGCTGAAATTCTACATCTTCCG

CAGCGACGAATCCGCAAACCTGACCCAAGGCTTTATGTACTTCAACGTTAATCAAACCATCCAGGAAGTG

GAAAATGTTGACCTGAGCGAGTTCATGCGCCGCATCAGCGGCAGCGAAATTGTTTTTCTGTTTAGCCTGT

TTGGTTTCGTCTGGCTGTTGCGTAAACACAAATCCATGATTATGGCACTGCCGATCTTGGTTCTGGGCTT

TCTGGCTCTGAAGGGCGGTCTGCGTTTCACCATCTACAGCGTGCCGGTGATGGCGTTGGGTTTTGGTTTC

CTGTTGAGCGAGTTTAAGGCGATTATGGTGAAAAAGTATAGCCAACTGACGTCGAATGTCTGCATCGTCT

TTGCCACCATTCTGACGCTGGCCCCAGTGTTCATTCATATCTACAACTACAAGGCACCGACCGTGTTTTC

GCAGAATGAAGCGTCTTTGCTGAACCAATTGAAAAACATCGCGAATCGCGAGGACTATGTCGTGACCTGG

TGGGACTATGGTTACCCTGTTCGTTACTATAGCGACGTCAAGACCCTGGTTGACGGTGGCAAACACCTGG

GTAAAGATAACTTCTTCCCGAGCTTTGCTCTGAGCAAAGATGAACAAGCGGCTGCCAATATGGCGCGTCT

GAGCGTCGAGTACACCGAGAAATCGTTCTACGCTCCGCAAAATGACATTCTGAAAACGGACATTCTGCAG

GCGATGATGAAAGACTATAACCAATCTAATGTCGATCTGTTCTTGGCCAGCCTGAGCAAGCCGGACTTCA

AGATTGATACTCCGAAAACGCGTGATATCTACCTGTACATGCCTGCACGTATGAGCCTGATTTTCAGCAC

CGTCGCAAGCTTTAGCTTCATTAACCTGGATACGGGTGTTCTGGACAAGCCGTTTACCTTCTCTACCGCC

TATCCGCTGGACGTGAAAAACGGCGAAATCTATCTGAGCAATGGCGTCGTCCTGAGCGACGATTTTCGTA

GCTTTAAGATCGGCGACAATGTGGTTTCTGTGAACAGCATTGTTGAGATCAATAGCATCAAACAGGGCGA

GTACAAGATTACGCCGATTGACGACAAGGCACAGTTCTACATTTTCTATCTGAAGGATAGCGCGATTCCG

TATGCGCAGTTCATCCTGATGGACAAAACGATGTTCAATAGCGCGTATGTTCAAATGTTCTTTCTGGGCA

ACTACGATAAGAACCTGTTCGACCTGGTGATCAACAGCCGTGATGCGAAAGTGTTTAAGCTGAAGATCTG

A

Figure 2: Screen shots from GeneDesigner software, showing the different ways the first 10 amino acids in the *pglB* gene could be coded. Top screenshot is the current existing sequence. Green is acceptable codons, red is for codons with usage in *E. coli* below 10%. The bottom image is for the suggested sequence and the codons recommended for *E. coli* after the optimisation are highlighted blue.

Primer sequences

WecA-Forward: TATATCTAGATTAGGAGGTATATATCGTGAATTTACTGACAGTGAG

WecA-Reverse: TATACCCGGGCTAATTAGGGGGTGTCGCCCTTGGGGTTATTTGGTTAAATTGGGGC

BacA-Forward: TATATCTAGATTAGGAGGTATATATCATGAGCGATATGCACTCGCT

BacA- Reverse: TATAGGATCCTTAAAAGAACACGACATACACC

pglB- Forward: TATAAGCGCTCACAATTCCACAACGGTTTCCCTCTAGA TTAGGAGGTATATATCATGTTGAAAAAAGAGTATTT

pglB- Reverse: TATAGGATCCTTAAATTTTAAGTTTAAAAAC

Restriction sites are underlined (XbaI TCTAGA, XmaI CCCGGG,BamHI GGATCC, AfeI AGCGCT).

pSRM targets

m/z 396.2

Total ion count for m/z 396.2

MS/MS spectra for compound circled above, elution time 28.6 min. The arrows below indicate transition ions.

m/z 871.5

Total ion count for m/z 871.5

MS/MS spectra for compound circled above, elution time 36.4 min. The arrows below indicate transition ions.

m/z 1041.4

Total ion count for m/z 1041.4

MS/MS spectra for compound circled above, elution time 39.8 min. The arrows below indicate transition ions.

m/z 927.7

Total ion count for m/z 927.7

MS/MS spectra for compound circled above, elution time 23.6 min. The arrows below indicate transition ions.

Growth data for engineered and control strains


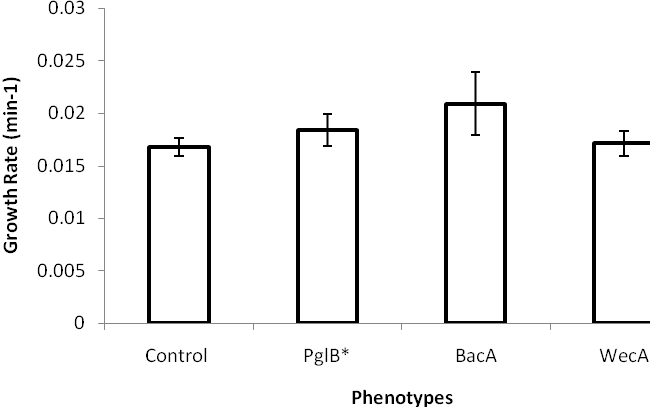

Supplement: Supplementary data 1 — The document containing supplementary material. [file mmc1.doc]
